# Do we know whether potential G-quadruplexes actually form in long functional RNA molecules?

**DOI:** 10.1042/BST20160109

**Published:** 2016-12-02

**Authors:** Carika Weldon, Ian C. Eperon, Cyril Dominguez

**Affiliations:** Leicester Institute of Structural and Chemical Biology and Department of Molecular and Cellular Biology, University of Leicester, Lancaster Road, Leicester LE1 9HN, U.K.

**Keywords:** biophysical methods, G-quadruplex, RNA biology

## Abstract

The roles of deoxyribonucleic acid (DNA) G-quadruplex structures in gene expression and telomere maintenance have been well characterized. Recent results suggest that such structures could also play pivotal roles in ribonucleic acid (RNA) biology, such as splicing or translation regulation. However, it has been difficult to show that RNA G-quadruplexes (G4s) exist in specific long RNA sequences, such as precursor messenger RNA, in a functional or cellular context. Most current methods for identifying G4s involve the use of short, purified RNA sequences *in vitro*, in the absence of competition with secondary structures or protein binding. Therefore, novel methods need to be developed to allow the characterization of G4s in long functional RNAs and in a cellular context. This need has in part been met by our recent development of a method based on a comparison of RNA and 7-deaza-RNA that provides a test for identifying RNA G4s in such conditions.

## Introduction

It is well established now that guanine-rich (G-rich) DNA sequences are able to form four-stranded secondary structures known as G-quadruplexes (G4s) (reviewed in refs [1,2]). G4s were first identified in DNA [3–5], and since then extensive investigations into DNA G4s have significantly improved our understanding of how they form, how they are stabilized, their various conformations and their impact on biological functions, where they are thought to function as biological switches, mainly in telomeres and promoter regions of oncogenes (reviewed in refs [6,7]). DNA G4s have been extensively studied *in vitro* and their existence *in vivo* has long been controversial [8]. However, the design of G4-specific antibodies has allowed direct observation of DNA G4s in cells [9,10].

## G-quadruplex structures

**Figure 1.**
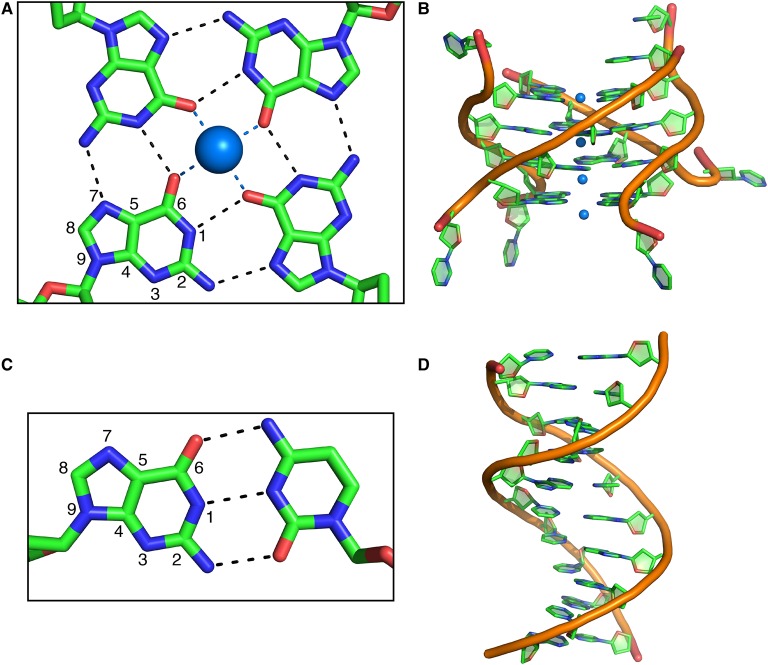
Comparison of G-quadruplex and helix structures. (**A**) G4 involves Hoogsteen base pair of guanines and is stabilized by cations. (**B**) Structure of a parallel G4 (PDB: 244D). (**C**) Watson–Crick base pair involving a guanine and a cytosine. (**D**) Structure of a DNA double-helix (PDB: 1BNA).

Unlike stem loops, which involve Watson–Crick base-pairing, G4s are stacked planes of G-quartets that involve Hoogsteen base-pairing (Figure 1) [5,11,12]. Structural studies on oligonucleotides have shown that each guanine forms four hydrogen bonds with two other guanines, which involve the atoms N1, N2, O6, and N7 (Figure 1A). These planes form large π-surfaces and thus π–π stacking stabilizes the structure. They are further stabilized by monovalent cations such as potassium or sodium [5]. G4s can be tetramolecular, bimolecular, or unimolecular and each strand can be in either the 5′-to-3′ or the 3′-to-5′ direction. Thus, many different conformations of G4s have been reported in DNA, but they are generally classified as parallel, when all strands are oriented in the same direction, antiparallel, when two are oriented in the opposite direction of the other two, or mixed, when one strand is oriented in the opposite direction to the other three [12]. The range of possible topologies is strongly dependent on the nature and the size of the loops connecting the G4-forming guanines [13,14]. Interestingly, in contrast with DNA, the ribonucleic acid (RNA) G4s that have been studied in small model systems can only adopt a parallel conformation, due to the conformational constraint exerted by the 2′-OH of the ribonucleotides.

## G4 in RNA biology

Although G4s have been well characterized in DNA, studies showing convincing evidence of their existence and biological importance in RNA are still limited [15,16]. RNA G4s can be observed in the cytoplasm of human cells [17], and the single-stranded nature of RNA molecules makes them more prone to forming G4s. There is evidence that G4s do exist in telomeric RNA [18,19], and G4s have also been invoked in studies on translation initiation [20–22], 3′-end processing [23,24], and alternative splicing [25–32].

## Current methods for identifying and characterizing RNA G4s

Current common strategies for determining the presence of G4s include: (1) identifying G4-forming sequences by using bioinformatics predictive tools; (2) making synthetic DNA or RNA oligonucleotides containing the putative G4-forming sequence and performing various biophysical studies; (3) determining the importance of the nucleotides involved by site-directed mutagenesis; and (4) using G4-stabilizing ligands to observe changes in functional assays.

### Bioinformatic approaches

**Figure 2.**
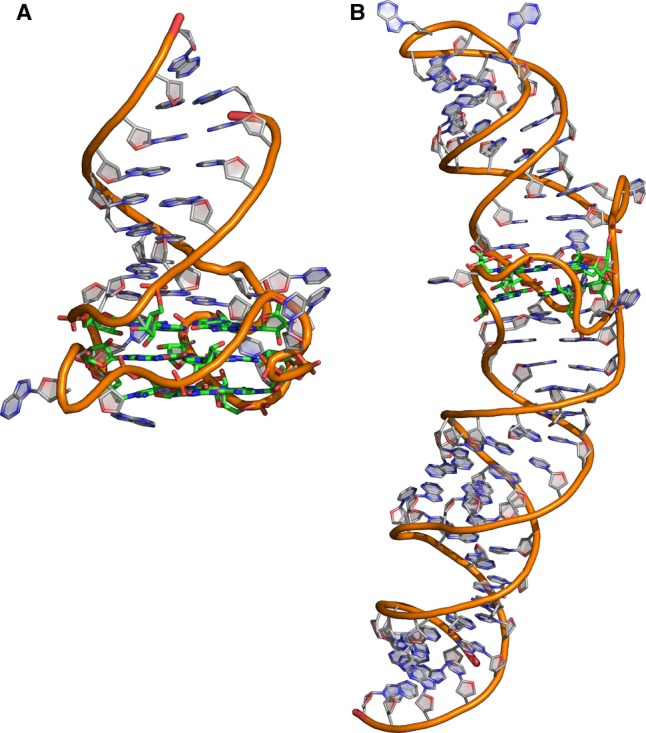
Non-canonical RNA G4s. (**A**) Structure of the Sc1 RNA duplex–quadruplex (PDB: 2LA5). (**B**) Structure of the Spinach aptamer (PDB: 4KDZ). The G4 is displayed in green and the duplex in grey.

Bioinformatic approaches to determine the secondary structure of nucleic acids have been established for many decades [33]. However, the tools commonly used to predict RNA secondary structures, such as Mfold [34], do not have the ability to take G4s into account. Therefore, other bioinformatic tools dedicated to G4s, such as QGRS Mapper, or QuadParser, have been developed [35–37]. These tools can estimate G4s with two or three tetrads with the following motif: G*x*N*y*_1_G*x*N*y*_2_G*x*N*y*_3_G*x*, where ‘*x*’ is the number of guanines (*x* > 1) and ‘*y*_1_, *y*_2_, and *y*_3_, are the lengths of the loops (*y* < 37). While this consensus sequence has allowed the identification of many biologically relevant DNA G4s, it might not be appropriate for RNA G4s [38]. RNA molecules being single-stranded, they display a much larger structural diversity than DNA, often involving long-range interactions, and RNA G4s might be embedded within larger structural motifs. Accordingly, structural studies have identified and characterized such RNA G4s, whose consensus sequences differ significantly from the consensus. For example, the *sc1* RNA bound by the RGG domain of FMRP adopts a duplex–quadruplex structure and the G4-forming sequence is G-N_2_-G-N-G_2_-N_2_-G_2_-N-G-N-G_2_-N_2_-G_3_ [39] (Figure 2A). Similarly, the SPINACH aptamer contains a G4 composed of two tetrad stacks that are embedded within two coaxially stacked helical stems and whose sequence is G_2_-N_2_-G_2_-N_26–35_-G-N_2_-G-N-G-N-G [40,41] (Figure 2B). Furthermore, the high stability of the consensus DNA G4 sequence might not be appropriate if an RNA G4 is part of a *cis*-acting regulatory sequence that requires a conformational switch for its function. It is therefore possible that less-stable RNA G4s exist and play significant biological roles in RNA processing, or other processes in the cell. These examples might explain why RNA G4s are less well characterized than their DNA counterpart and strongly suggest that new bioinformatics tools must be developed for the identification of RNA G4s [42].

### Biophysical approaches with short sequences

Once a potential G4 has been identified by bioinformatics tools, the G4 has in most studies been characterized *in vitro* using various biophysical methods with short sequences in isolation from the rest of the RNA. These studies usually include circular dichroism (CD) [43], ultraviolet (UV) melting curves [44], isothermal titration calorimetry (ITC) [45], and nuclear magnetic resonance (NMR) spectroscopy [46]. Furthermore, since G4s are strongly stabilized by potassium (K^+^), but not by lithium (Li^+^) ions [5], G4 biophysical characterizations are often done by comparing the biophysical signals of a putative G4 in buffers containing either K^+^ or Li^+^.

**Figure 3.**
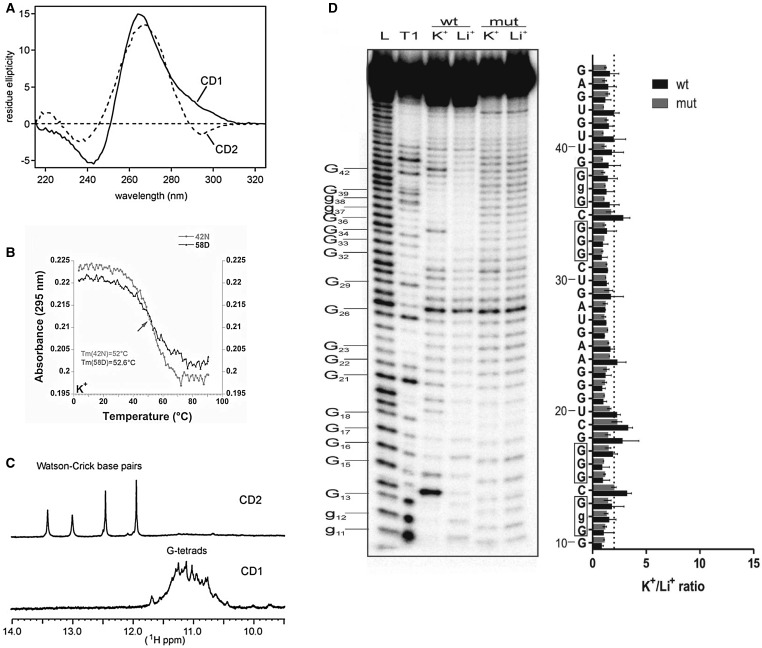
Biophysical and biochemical characterization of G4s. (**A**) CD spectrum of G4 (CD1) and stem-loop (CD2) RNAs (reproduced with permission from ref. [32]). (**B**) UV melting profile showing the absorbance at 295 nm as a function of the sample temperature of two RNA G4 sequences (reproduced with permission from ref. [30]). (**C**) ^1^H NMR spectra showing the imino regions of G4 (CD1) and stem-loop (CD2) RNAs (reproduced with permission from ref. [32]). (**D**) In-line probing of a 54-nucleotide G4 RNA in the presence of either K^+^ or Li^+^ ions (reproduced with permission from ref. [38]).

CD has been widely used to characterize G4s. The CD spectrum differs significantly depending on the topology of the G4. Antiparallel G4s typically display a spectrum with a positive signal at 295 nm and a negative signal ∼260 nm. In contrast, parallel G4s, which include all RNA G4s, typically display a CD spectrum with a positive signal at 265 nm and a negative signal at 240 nm [43,47]. Unfortunately, RNA sequences adopting an A-form helix display a very similar CD signature to that of parallel G4s and it is difficult to differentiate G4 from stem-loop structures in RNA (Figure 3A). To circumvent this issue, CD spectra are typically measured in two different buffers that contain either K^+^ or Li^+^. A difference in signal intensity at 265 and 240 nm can then be attributed to G4 formation since stem-loop formation is not dependent on the presence of K^+^.

UV measurements of nucleic acids are characterized by an absorbance peak at 260 nm, which does not allow for the discrimination of G4 from other putative structures. However, G4s have a unique hypochromic signature at 295 nm [44]. Therefore, G4 characterization by UV can be done by measuring the absorbance at 295 nm as a function of the sample temperature, leading to a hypochromic melting transition and the determination of the G4 melting temperature that is indicative of the stability of the G4 in a certain buffer (Figure 3B).

NMR, like X-ray crystallography, has been extensively used to obtain high-resolution structures of DNA and RNA G4s [48]. Indeed, out of 184 G4 structures that have been deposited in the protein databank (PDB), 112 were solved by NMR spectroscopy. Furthermore, although full structure determination of G4s is a lengthy process, NMR experiments can also be used at an early stage to characterize putative G4s and rapidly provide low-resolution information that is complementary to CD and UV [46]. Of importance, NMR spectra of nucleic acids display typical signals ∼10–15 ppm that arise from the imino protons of guanine, thymine, or uracil. If these protons are not hydrogen-bonded, they exchange with the solvent and become invisible in NMR spectra. However, if these protons are involved in hydrogen bond, as a consequence of Watson–Crick or Hoogsteen base-pairing, they become protected and visible. It is therefore possible to easily identify DNA or RNA structures using one-dimensional NMR. Furthermore, the resonance frequency of iminos in G4s is typically ∼10–12 ppm, which is different from iminos involved in Watson–Crick base-pairing (12–15 ppm) (Figure 3C). NMR can therefore be used to discriminate between G4 and helix structure formation of nucleic acids. In addition, because each imino proton leads to one peak, it is possible to estimate whether the G4 adopts single or multiple conformations as well as the number of guanines that contribute to the G4 structure.

Although ITC is mainly used to investigate the thermodynamic properties of biological complexes and has been used extensively to study the interaction of small molecules with G4s, it can also provide information on the thermodynamics of G4 folding [45]. This can be achieved by titrating a solution of K^+^ into a solution containing a putative G4 oligonucleotide. The gradual addition of K^+^ induces the formation of G4 that produces a heat change measured by ITC, providing valuable information on the enthalpy of G4 formation [49].

While these studies are definitive and give a strong indication that the DNA or RNA studied can form a G4, they are generally performed on short nucleic acid sequences that do not compete with alternative secondary structures, which are complex and abundant in RNAs; therefore, they remain outside of the physiological context of the RNA under study.

## Biochemical methods

Biochemical methods focus on the secondary structure determination of the RNA in conditions that either favour (presence of K^+^ ions) or disrupt (presence of Li^+^ ions) the G4 structure (Figure 3D) [21,50]. Most of these methods have only been used on short RNA segments rather than long functional RNA transcripts. However, reverse transcriptase pauses at G4 structures, and thus by using internal primers, K^+^-dependent pausing can provide evidence for G4s within long RNA sequences, even in complex mixtures of RNA [51]. It may be possible to extend this to a genome-wide analysis, although it will be difficult to do so without an RNA fragmentation step. However, the requirement of comparing G4 formation in different buffers prevents the use of these methods directly in physiological environments such as nuclear or cell extracts. Pausing of reverse transcriptase at G4s has been detected in nuclear extracts, although in such cases other evidence is still required to show that the pause was caused by a G4 [31].

## Functional approaches

The functional significance of G4s that have been identified by bioinformatics studies and characterized by biophysical approaches is generally further investigated by site-directed mutagenesis of the guanines involved in G4 formation together with a functional assay. In the case of RNA, functional assays include translation using reporter genes such as luciferase [21,52] or alternative splicing assays [25,29,32]. While this method certainly suggests the possibility that a specific G4 is functional, such data should be analyzed with care, since the change in functional response induced by the mutation could also be due to other effects, such as an alteration in the protein-binding pattern or secondary structure of the RNA. For example, mutation of a guanine in RNA could prevent the binding of the splicing factors hnRNP F/H, which specifically bind G-rich sequences [53].

More than 800 small molecules have been described to bind specifically to G4 DNA and/or RNAs [54]. An alternative to site-directed mutagenesis of guanines therefore consists of evaluating the effect of such ligands in functional assays [25,26,29–31]. It is generally believed that all G4 binders act as stabilizers of G4s; therefore, the effect observed upon the addition of a ligand in functional assay can be attributed to the effect of the G4. However, some ligands are not very specific to G4s and the observed outcome might arise from other effects; moreover, it is unclear whether all these molecules are G4 stabilizers. For example, the well-characterized G4 stabilizer, TMPyP4, has also been shown to destabilize some RNA G4s [55]. Furthermore, although these ligands may be binding to a G4 and thus exerting an effect, there is no evidence that the G4 exists naturally in the absence of the G4 ligand. Like antibodies, the G4 ligand could be inducing the formation of a G4 that would otherwise not exist.

## Future challenges for investigating cellular and functional RNA G4s

Although there have been many suggestions that RNA G4s exist and have roles in RNA processing or other cellular processes, a clear demonstration of either claim is still lacking. All the approaches used to study RNA G4s have been adapted from extensive studies of DNA G4s. However, in contrast with DNA, RNA is single-stranded and other secondary structures are also highly likely to form that can compete with G4 formation. It is therefore essential to investigate RNA G4s in the context of their physiological sequence, rather than using short RNA regions. It will be very important to find ways of generating high-throughput information derived from full-length RNA in cellular conditions. The identification of putative G4s relies mainly on bioinformatics tools that predict the probability of a query sequence to form a G4 based on pre-defined G4 consensus sequences. While such tools estimated the presence of >300 000 putative G4 in cellular RNAs, recent studies have demonstrated that, in contrast with DNA, RNA G4s can adopt more complex structures embedded within a larger and complex structural context and that the DNA consensus G4 sequence is probably not adequate to appreciate the full structural diversity of RNA G4s (Figure 2). It is therefore essential to develop novel bioinformatics tools that can evaluate with a higher accuracy the potential of a sequence to form a G4 [42]. Powerful bioinformatics tools exist to predict either RNA secondary structure in long functional RNAs [34] or the formation of G4s [35–37], but to date no tools have been described that can simultaneously take into account the coexistence of secondary structures and G4s within a long RNA molecule. Such tools would be instrumental for predicting more accurately the possible RNA structures and to help in the design of experiments that address the functional relevance of G4s in functional RNAs. However, of course, the development of such tools will require extensive further structural characterizations of RNA G4s in long functional RNAs.

The major challenge in the RNA G4 field remains the investigation of such structures in cellular environment. Using a specific G4 antibody, RNA G4s have been observed in cells, proving their existence in a cellular context [17]. However, as described earlier, most approaches to characterizing G4 RNAs rely on a comparison of the RNA sequence in the presence of K^+^ or Li^+^ ions, preventing the investigation of G4s in a cellular context. The best method available that can be applicable in long RNA and in physiological conditions is the use of a G4-stabilizing ligand to observe functional changes in functional assays. The major advantage is that such experiments can be done in cells. However, by using a G4 ligand, there arises another area of ambiguity; does the G4 form naturally or has the G4 ligand caused the G4 to form? This is also the case for the G4 antibody [17], as addition of the antibody may shift the equilibrium of the RNA population to the G4-containing form. The possibility of this is emphasized by our previous work, showing that a short oligonucleotide that acts as a *trans*-acting enhancer of RNA splicing exists as a diverse population of molecules under functional conditions, bound as a linear sequence by various sets of mutually incompatible proteins and also a G4 [31]. The possibility that RNA structures are not static but in protein-augmented dynamic equilibria adds to the complexity of the challenge in studying G4s. Therefore, novel methods must be developed to allow the characterization of G4s in a cellular context without interfering with the conformational space of the RNA.

We have recently developed a novel strategy to identify G4s in long RNAs in a functional context [56]. This strategy relies on the different hydrogen-binding pattern of G4s and stem-loop structures (Figure 1). Substitution of guanines by 7-deaza-guanines, in which the nitrogen at position 7, N7, is replaced by a carbon, prevents Hoogsteen base-pairing and G4 formation, but does not affect the formation of Watson–Crick base-pairing [57]. By identifying the differences between an unmodified RNA of 681 nucleotides and its deazaguanine substituted analogue, using ribonuclease (RNAse) footprinting and RNAse H digestion patterns in nuclear extracts, we are able to provide evidence for the presence and position of G4s in relatively long functional RNAs and in functional conditions. The method may not work with RNA molecules containing strong tertiary structures, but it is likely to enable G4 mapping in specific precursor messenger RNA molecules. The future, however, will belong to the high-throughput methods yet to be invented.

## Abbreviations

CD: circular dichroism
DNA: deoxyribonucleic acid
G4: G-quadruplex
G-rich: guanine-rich sequence
ITC: isothermal titration calorimetry
K^+^: potassium ion
Li^+^: lithium ion
NMR: nuclear magnetic resonance
PDB: protein databank
RNA: ribonucleic acid
RNAse: ribonuclease
UV: ultraviolet
